# The Antioxidant, Anti-Inflammatory, Pathological, and Behavioural Effects of *Medicago sativa L*. (Alfalfa) Extract on Brain Injury Caused by Nicotine in Male Rats

**DOI:** 10.1155/2021/6694629

**Published:** 2021-03-05

**Authors:** M. Raeeszadeh, P. Mortazavi, R. Atashin-Sadafi

**Affiliations:** ^1^Department of Basic Sciences, Sanandaj Branch, Islamic Azad University, Sanandaj, Iran; ^2^Department of Pathobiology Sciences, Science and Research Branch, Islamic Azad University, Tehran, Iran; ^3^Graduate of Faculty of Veterinary Sciences, Sanandaj Branch, Islamic Azad University, Sanandaj, Iran

## Abstract

Nicotine is one of the most important compounds in cigarette which can cause changes in the concentration of neurotransmitters and damage to the nervous system. The aim of this study was to investigate the effect of the hydroalcoholic extract of *Medicago Sativa L*. (alfalfa) on controlling nicotine-induced brain damage and anxiety behaviour in rats. Forty-two male Wistar rats were randomly divided into six equal groups and treated daily as follows: a control group, T1 and T2 groups where animals were subcutaneously injected 250 and 500 mg/kg alfalfa extract, respectively, T3 and T4 groups where animals were injected subcutaneously 0.2 mg/kg nicotine and 250 and 500 mg/kg alfalfa extract, and T5 group in which only nicotine at the dose of 0.2 mg/kg was injected. At the end of the period after weighing, the elevated plus-maze test was taken from the animals. Serum assay was conducted to measure TCA, IL-1, and TNF*α*, and half of the brain tissue was used to measure oxidative stress parameters (GPx, SOD, TAC, and MDA) and the other parts were used for histopathological studies. Body weight in the T5 group was significantly different from that of the other groups. The time and number of open arms reduced in the T5 group. The duration and number of times in the open arm significantly decreased in the treated groups in a dose-depended manner. Malondialdehyde concentration was the highest in the nicotine group and the lowest in T2. The concentration of GPx and SOD was significantly increased in the presence of alfalfa extract in nicotine groups. TNF*α* and IL-1 in the T5 group showed a significant increase compared to the other groups. Moreover, the number of neurons and the level of necrotic neurons and gliosis significantly decreased and increased in the nicotine group, respectively, while these histopathological damages improved by treatment with alfalfa extract in T3 and T4 groups. Alfalfa extract can have a significant dose-dependent therapeutic effect on inducing oxidative damage and inflammatory responses of nicotine in the brain and reducing anxiety behaviours.

## 1. Introduction

Cigarettes contain more than 7400 toxins, of which nicotine is one of the most important. Nicotine has been shown to have effects on anxiety and depression in both human and animal studies. Anxiety is a condition that every person experiences in life. Decreasing the synaptic threshold during anxiety has been shown to increase the defensive response to normal stimuli [1].

Different effects of nicotine on various organs including the ovaries [2], lungs [3], pancreas [4], and brain and brain cells [5] have been reported in hamsters, rats, and mice [6]. Nicotine stimulates the release of the chemical transporters glutamate, GABA, acetylcholine, dopamine, norepinephrine, and serotonin [7].

Nicotine is also one of the compounds that induce intracellular oxidative stress and is known as an important factor in the damage of biological molecules. Previous experiments using animal models and cell culture have shown that relatively high concentrations of some ROS, such as NO and H_2_O_2_, can act as signal transducers. Nuclear transcription factor-kappa B (NF-kappa B) and TNF-*α* are involved in many biological processes such as inflammation, innate immunity, and apoptosis [8]. Previous studies have not found a comprehensive consensus on the effects of nicotine on anxiety as an anxiolytic or anxiety agent. It can be due to differences in species, race, nicotine content, dose of nicotine, and type of anxiety test [9]. Among these, the dose of the drug is more important. Studies have shown that low levels of nicotine have anxiolytic effects while its high levels are stressors [10].

Benzodiazepines and barbiturates are used to treat anxiety. However, due to the harmful effects of these chemical drugs, efforts have been made to find less harmful sedatives and antianxiety drugs, and in this regard, the production of plant-based drugs has expanded. In recent years, several benzodiazepines and similar ligands have been identified in natural sources and medicinal plants. For example, flavonoids with similar function to benzodiazepines and phytoestrogens have been extracted from chamomile [11, 12]. Flavones in plant foods have estrogenic powers called phytoestrogens. Another study showed the anxiolytic effects of phytoestrogens and attributed the cause to a change in the GABA receptor or its effect [12].

Phytoestrogens are natural compounds found in plants. Isoflavones, comstans, and lignans are three important subsets of phytoestrogens. Isoflavones are a subset of estrogens. Isoflavones, which have been studied more than other phytoestrogens, have mammalian estrogen-like properties [13]. Isoflavones are found in large amounts in soy and its products, as well as in the *Medicago sativa L*. (alfalfa) plant.

Due to the importance of phytoestrogenic and antioxidant compounds in alfalfa and since there was no previous study analyzing their effects in controlling the anxiolytic effects of nicotine, the aim of this study was to evaluate different doses of hydroalcoholic extract of alfalfa and nicotine separately and simultaneously in anxiety behaviors and changes in oxidative and pathological stress markers in the brain.

## 2. Materials and Methods

Forty-two male Wistar rats weighing 200–250 gram were selected. The animals were fed with healthy water and plates at standard conditions of light, temperature, and humidity for one week for adaptation. In the present study, the environmental conditions, use, and euthanasia of animals were considered according to the international ethical guidelines for laboratory animals. Ethical code was obtained from Kurdistan University of Medical Sciences (IR.MUK.REC.1398.167).

Animals were randomly divided into six equal groups and treated daily as follows:

The control group received normal saline intraperitoneally.

  T1 group: animals received hydroalcoholic extract of alfalfa in a dose of 250 mg/kg.
  T2 group: animals received hydroalcoholic extract of alfalfa in a dose of 500 mg/kg.
  T3 group: animals received hydroalcoholic extract of alfalfa in a dose of 250 mg/kg intraperitoneally with nicotine in a dose of 0.2 mg/kg.
  T4 group: animals received hydroalcoholic extract of alfalfa in a dose of 500 mg/kg intraperitoneally with nicotine in a dose of 0.2 mg/kg.
  T5 group: animals received nicotine tartrate hydrogen salt (Sigma-Aldrich company Lot Number = 029H046) with a concentration of 0.01% in a dose of 0.2 mg/kg injection subcutaneously for 25 days. On day 26, the elevated plus-maze test as one of the tests used to identify the anxiogenic and anxiolytic effects of drugs was used to measure anxiety [14]. This test is based on the work of Pellow and File (1986), which is designed based on two instincts: searching sense of rodents and avoiding open and bright environments is another part.

This tool is made of wood and in the shape of plus (+) which is mounted on a base of 40 cm from the ground. Its arm sizes (70 × 40) are completely standard. The amount of exposure in all arms is regulated through a lamp that is 100 watts placed on top of it at a distance of 140 cm. In this test, the number of times the animal enters the open and closed arms and also the stopping time in both arms are measured in 5 minutes [14, 15].

After measuring the body weight of all animals, they were euthanized by overdose of intraperitoneal sodium thiopental and blood samples were then taken from the heart. Serum was separated to measure some biochemical parameters by using a centrifuge (Centrifuge 5415 R; Rotofix 32A, Germany) at room temperature with 3000 rpm for 10 min. The whole brain was also emptied and a part of it was used to measure the concentration of malondialdehyde (MDA), glutathione peroxidase (GPx), superoxide dismutase (SOD), and total antioxidant capacity (TAC), and another part was used to examine histopathological changes after fixation in 10% formalin and staining with hematoxylin and eosin.

### 2.1. Method of Preparation of Hydroalcoholic Extract of Alfalfa

To prepare alfalfa extract, first, alfalfa plant was obtained from Ahvaz farms and the identity of this plant (No. 1856) was confirmed by the Herbarium Center of the University of Kurdistan and was placed in a dark place for 72 hours to dry. The leaves and flowers of the plant were pulverized by using the mill. Then, 150 g of dried powder was mixed with 75% ethanol to become one liter for 48 hours with a shaker and was filtered through a sieve. Finally, it was evaporated using a rotary vacuum of alcohol, and the extract was used as a lyophilized powder [16].

### 2.2. Method for Measuring Oxidative Stress Parameters

Brain tissue was used to determine the amount of MDA, which is the end product of lipid peroxidation using Satoh's method [17]. 1.5 ml of 10% trichloroacetic acid was added to 500 *μ*l of homogeneous tissue and centrifuged for 10 minutes. Then, 1.5 ml of the supernatant solution was removed, 2 ml of 0.67% thiobarbituric acid was added, and it was kept in Ben Marie at 100° for 30 minutes. Next, 2 ml of butanol was added, and after severe vortex, it was centrifuged for 15 minutes at 4000*g*. Then, the pink supernatant was read at 532 nm. Malondialdehyde concentration was determined using 1, 1, 3, and 3 tetraethoxypropane as standard, and it was calculated in nanomoles per mg. The standard solution of malondialdehyde is prepared at concentrations of 0.2–2 *μ*M in 10% sulfuric acid [17]. Measurement of oxidative stress enzymes on brain tissue extract was performed using the Zellbio-German kit method. Glutathione peroxidase was measured by Paglia and Valentine's method. In this method, glutathione peroxidation catalyses the oxidation reaction of glutathione and the reduction of hydroperoxide. Afterwards, NADP + was measured at 340 nm [18]. The concentration of superoxide dismutase enzyme is related to the function and ability of oxidizing oxygen ions O_2_^−^ [19, 20].

### 2.3. Measurement of Serum Antioxidant Capacity and Proinflammatory Cytokines

Total antioxidant capacity was assessed by Benzie and Strain using the FRAP method [21]. In this method, the ability of serum to reduce ferric ions is measured [19]. Serum concentrations of Tumor necrosis factor alpha (TNF*α*) and interleukin-1 (IL-1) as proinflammatory cytokines were measured by ELISA using commercial kits (R&D Systems, Minneapolis, USA) [22].

### 2.4. Histopathological Studies of the Brain

Neuronal necrosis and gliosis were measured based on quantitative pathological criteria in sections prepared from the animal brain. Also, the neuronal number was counted in the frontal cortex in five 10X fields (Analysis by Image J software).

### 2.5. Data Analysis

Data from the study were reported as mean ± standard error (Mean ± SEM). To compare the mean of parametric data between groups, one-way ANOVA and Tukey's test were used, and for the nonparametric data, the Mann–Whitney *U*-test was used. All statistical analyses of the data were performed by SPSS 23, and *P* < 0.05 was considered as a significant level.

## 3. Results

**Figure 1:**
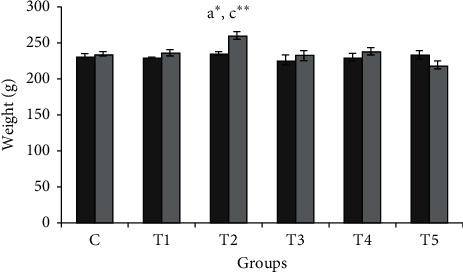
Animal body weight values on the first and last day of the study in different groups. Each column represents the mean ± SEM. ^*∗*^*P* < 0.05 and ^*∗∗*^*P* < 0.01 represent a and c, respectively, compared to the C and T2 groups.

Regarding the weight of the studied animals, at first, there was no statistically significant difference between the weights of the animals in different groups. On the last day of the study, the highest weight was 260.5 ± 5.83 in the T2 group and the lowest was 219.5 ± 5.15 in the T5 group. There was a statistically significant difference in the body weight of animals in the T5 group compared with the T2 group, but there was no statistical difference compared with T1, T3, and T4 groups. No statistically significant difference was observed in the weight of animals in the control group compared with T1, T3, and T4 groups (Figure 1).

The elevated plus-maze test is one of the standard tests to measure the degree of anxiogenic and anxiolytic behavior to herbal or chemical combinations. In this study, the control and experimental groups underwent behavioral anxiety tests before performing biochemical tests. In this test, the shortest stay time in the open arm belonged to the T5 group with 90.42 ± 8.27 and the longest time was in the T2 group with 150.78 ± 6.34 seconds. A significant increase in time was observed compared to the control group with T1 and T2 groups. Regarding the number of entries in open arms in T2 and T4 groups, a significant difference was seen with the control group. The reduction of time spent and the number of entries in open arms in the T5 groups was compared to other groups (*P* < 0.05) (Table 1).

The highest duration and number of times spent in the closed arm were seen in the T5 group, and the lowest were seen in the T2 group. Treatment with alfalfa extract significantly reduced the retention time in the closed arm in T1, T2, T3, and T4 groups (Table 2).

**Figure 2:**
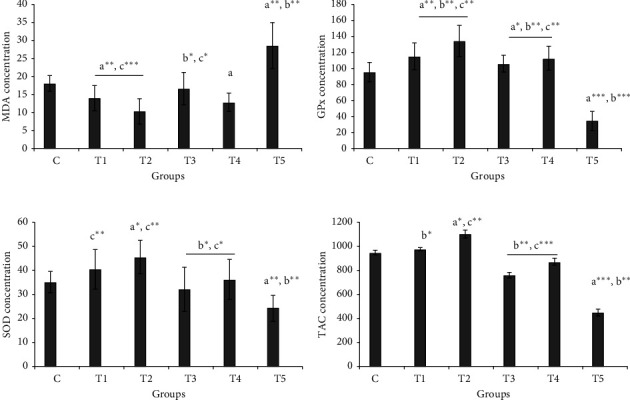
Stress oxidative parameters in different groups. (a) Brain malondialdehyde (MDA) levels in different experimental groups. (b) Glutathione peroxidase (GPx) levels in brain tissue of various tested animals. (c) Concentration of the superoxide dismutase (SOD) enzyme. Total capacity antioxidant (TAC) concentration in different tested groups. Each column represents the mean ± standard. ^*∗*^*P* < 0.05, ^*∗∗*^*P* < 0.01, and ^*∗∗∗*^*P* < 0.001 represent a, b, c, and d, respectively, compared to the C, T1, T2, and T3 groups.

Changes in oxidative stress parameters in brain tissue were observed in control and experimental groups, and the highest concentration of malondialdehyde in brain tissue was obtained in the nicotine group. These values in the control, T1, T2, T3, T4, and T5 groups were 18.03 ± 2.1, 14.04 ± 3.4, 10.23 ± 3.3, 16.56 ± 4.56, 12.8 ± 2.4, and 28.54 ± 6.32 nmol/mg, respectively. The concentration of malondialdehyde in brain tissue in the group receiving nicotine (T5) was significantly higher than in the other groups (control, T1, T2, T3, and T4 groups), while it was the lowest in the T2 group when compared to the other groups (Figure 2(a)).

The amount of glutathione peroxidase (GPx) in the brain tissue reached 95.67 ± 12.45 U/mg in the control group, 134.65 ± 18.90 U/mg in the T2 group with the highest amount, and 34.89 ± 12.12 in the T5 group with the lowest amount. The amount of glutathione peroxidase in group 5 was significantly reduced compared to the control, T1, T2, T3, and T4 groups. There was a statistically significant difference between the concentrations of glutathione in brain tissue in the control group with other experimental groups (Figure 2(b)).

The amount of superoxide dismutase in brain tissue was the highest in the T2 group and the lowest in the T5 group. There was a statistically significant difference between the concentration of superoxide dismutase in brain tissue between the control group and the T5 group (*P* < 0.01). Decrease in the amount of SOD in the T5 group compared with the T2 group was significant but was nonsignificant compared with the T3 group (Figure 2(c)).

The highest concentration of total serum antioxidant capacity was seen in the T2 group 1100.89 ± 34.24 *μ*mol/ml which had statistically significant difference with the control group 950.45 ± 21.23 *μ*mol/ml; T3 group, 760.49 ± 23.56 *μ*mol/ml; T4 group, 870.53 ± 27.80 *μ*mol/ml; and T5 group 450.67 ± 23.45 *μ*mol/ml. A significant decrease was observed between T5 group with the control group and the other experimental groups (Figure 2(d)).

Regarding the levels of pro-inflammatory cytokines, IL-1 had the highest concentration in T5 group (198.47 ± 23.12), and T2 group with 76.54±14.29 pg/ml level was the lowest value. There was no statistically significant difference between the amount of IL-1 in the control group and the T1 and T2 groups. But, the T5 group showed a statistically significant difference with all experimental groups. When alfalfa extract was administered at a dose of 500 mg/kg, the serum IL-1 level showed a significant decrease compared to the nicotine group. Serum TNF*α* levels decreased in dose-dependent alfalfa groups, and this decrease was significant in the T2 group compared to the control group. The highest concentration of TNF*α* in the nicotine group was 253.56 ± 10.46 which was significant compared to the other groups (*P* < 0.001). The highest levels of proinflammatory cytokines were observed in the T5 group which had significant in comparison with the other groups (*P* < 0.05) (Table 3).

The highest level of neuronal damage and gliosis in brain tissue was seen in the T5 group, and the lowest damage was detected in the control, T1, and T2 groups. There was a significant difference between the T5 group and the control, T1, T2, and T4 groups (Table 4).

The lowest level of neurons in the cerebral cortex was detected in the T5 group (36.60 ± 6.78), and the highest rate was in the T2 group (82.49 ± 8.91) when compared to the other groups. However, treatment with alfalfa extract increased the level of neurons in T3 and T4 groups (Table 5).

**Figure 3:**
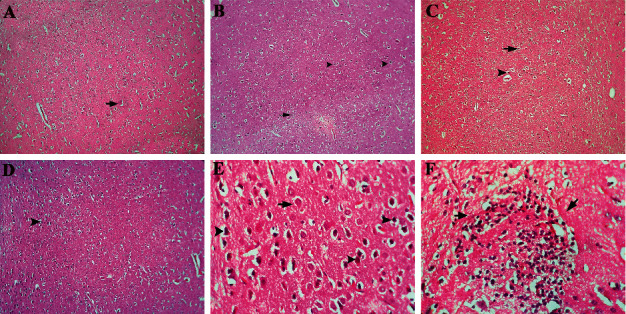
Histopatological study in the brain section of the animal studied. (a) (Control group): brain frontal cortex in the control group, a normal neuron (arrow) and neuroglia are seen. (b) (T3 group): brain frontal cortex in the T3 group, a severe red neuron (arrow head) that is an indication for neuronal necrosis and a few normal neurons are seen. (c) (T4 group): brain frontal cortex in the T4 group, a moderate red neuron (arrow head) that is an indication for neuronal necrosis and a normal neuron are seen. (d) (T5 group): brain frontal cortex in the T5 group, a severe red neuron (arrow head) that is an indication for neuronal necrosis is seen (H&E, 100x). (e) (T5 group): brain frontal cortex in the T5 group, a severe red neuron (arrow head) that is an indication for neuronal necrosis and a few normal neurons (arrow) are seen. (f) (T5 group): brain frontal cortex in the T5 group, focal gliosis (arrow) is seen (H&E, 400x).

As shown in Figure 3, red necrotic neurons increased in the T5 group, whereas the focal gliosis is observed in this group, while in T3 and T4 groups, because of administration extract, the number of necrotic neurons decreased (Figure 3).

## 4. Discussion

Due to the increasing use of cigarettes and tobacco and exposure to nicotine as one of their harmful products, the approach of using plant compounds to control the damage induced by them has been very welcomed. Therefore, the aim of this study was to investigate the effect of using alfalfa extract in different doses on brain tissue changes and nicotine-induced anxiety behaviors in male Wistar rats. The results of this study showed that nicotine significantly could reduce body weight, which is in agreement with a previous finding [23]. Reasons for this effect can be due to decreasing appetite, food intake, and calorie intake or increasing basal metabolism in the face of nicotine [24]. In groups treated with alfalfa extract, the body weight increased compared to the nicotine group. It can be due to increased food intake and decreased nutrient metabolism [25]. In a study on lambs, weight gain, weight change, and decrease in carcass fat during alfalfa treatment were reported due to the increased food intake and the increased diet quality [26]. In this regard, the findings of this study revealed that the combined groups treated with alfalfa and nicotine improved in weight, especially at a dose of 500 mg/kg.

The duration and number of times in the open arm in the nicotine group had a significant decline which indicated the anxiogenic effects of nicotine. The anxiogenic effects of nicotine are due to its effect on acetylcholine receptors, resulting in stress and depression in normal brain conditions [27]. Nicotine can also release neurotransmitters such as GABA, glutamate, serotonin, norepinephrine, and dopamine. According to previous studies, the anxiogenic and anxiolytic effects of nicotine depend on the duration and dose of nicotine used [27, 28]. The results of this study showed that nicotine at a dose of 0.2 mg/kg for 25 days could induce an anxiogenic effect that can be an acute intoxication. Increasing the duration of staying in the open arm and the number of times moving in the open arm in the treatment groups showed the sedative effects of the extract on the animal. Furthermore, the extract was also able to control nicotine-induced anxiety, which reduced stopping and movement in the open arm and increased the length of stay in the open arm.

In a study that investigated the anxiolytic effects of alcoholic alfalfa extract, it was found that these effects are similar to the anxiolytic properties of diazepam [29]. On the other hand, phytochemical compounds of flavonoids, phenolics, and tannins were considered as anxiolytic compounds in the control of diseases related to the nervous system [30, 31]. Changes in MDA concentration in brain tissue have been shown to be an oxidative stress factor and an indicator of fat peroxidation. Increased oxidative stress by nicotine directly increased damage to lipids and DNA and, ultimately, damage to the brain and changes in a person's mental balance [32, 33]. The level of MDA in a dose-dependent manner was decreased by alfalfa extract in treated groups, so that the lowest concentration of MDA was observed in the treated group with 500 mg/kg of extract. The results of this study also showed that the activity of GPx and SOD along with total antioxidant capacity decreased by nicotine, while the level of them increased with the administration of different doses of alfalfa extract in a dose-dependent manner. In the nicotine group, severe oxidative damage was observed, which caused significant damage to brain tissue. Consistent with this evidence, it has been reported that long-term use of high-dose nicotine can cause oxidative stress by producing ROS, and spirulina could inhibit this effect [34]. They also showed that *Spirulina platensis*, by antioxidant and anti-inflammatory actions, causes neuroreceptive effects against nicotine poisoning and neuronal damage. It is possible that nicotine activates the apoptotic pathways and causes brain damage by increasing the Nuclear Factor-kappa B (NF-*κ*B) pathway, a hypothesis supported by rising TNF*α* and IL-1 levels in our study. There is strong evidence that the NF-*κ*B pathway plays a key role in the expression of genes for proinflammatory cytokines and chemokines [34]. Under normal conditions, NF-*κ*B is locally released in the cytosol and inhibited by binding to the I*κ*B protein. When the level of internal and external antioxidants fails to eliminate ROS [34], the stimulation created by the oxidants can activate this pathway. The result will be an increase in the production of proinflammatory cytokines IL-1 and TNF*α* [35]. In the present study, the highest level of necrosis and gliosis and a significant decrease in the number of neurons were seen in the nicotine group. While treatment with alfalfa extract could decrease necrosis and increase the average number of neuron cells, the improvement of serum level of TNF*α* confirms these changes as well as this. Alfalfa has been shown to have an anti-inflammatory activity and can improve the damage caused by inflammation. Sadeghi et al. investigated the effect of alfalfa extract on oxidative damage induced by iron oxide nanoparticles in the rat liver. They showed that alfalfa extract can reduce oxidative stress in liver cells by increasing GPx levels, thereby reducing the level of liver enzymes and reducing DNA fragmentation [36]. Another study reported that saponins are one of the most important antioxidant compounds against phagocyte function and oxidative stress [37]. Shi et al. showed alfalfa saponins had a very high potential in inhibiting cell damage and cell death due to oxidants [37, 38]. Carotenoids and anthocyanins also showed antioxidant effects against single molecular oxygen and peroxide radicals [39]. Alfalfa extract is rich in these compounds. Therefore, it can be considered as a compound with multiple antioxidant effects. In order to confirm the effectiveness of alfalfa extract on oxidative damage induced by nicotine in the brain, the pathological results of brain tissue also confirmed these results, so that the degree of pathological damage in the 500 mg/kg alfalfa extract group showed a significant decrease compared to the nicotine group.

## 5. Conclusions

It can be concluded that subcutaneous injection of nicotine (0.2 mg/kg) could reduce body weight and cause oxidative stress, inflammation, and anxiety. These effects can be due to reduced time spent in the open arms, increased MDA, and decreased antioxidant enzymes in the brain. In addition, inflammation due to increased concentrations of proinflammatory cytokines can result in necrosis and gliosis. Alfalfa extract, due to the presence of polyphenolic antioxidant compounds, was able to reduce nicotine brain damage and improve oxidative and inflammatory status. Therefore, alfalfa can be useful in reducing the side effects of diseases related to inflammation and oxidative stress such as anxiety in the new century.

## Figures and Tables

**Table 1:** Changes in the time spent and frequency of entry into the open arms.

Groups	Time spent in open arms (sec)	Number of entries in open arms
C	125 ± 9.12	4.02 ± 1.4
T1	140.32 ± 5.35 a^*∗*^	5.7 ± 0.3
T2	150.78 ± 6.34 a^*∗∗*^	6.8 ± 2.3 a^*∗*^
T3	129.1 ± 8.43 b^*∗*^, c^*∗∗*^	3.4 ± 1.9 c^*∗*^
T4	141.97 ± 4.123 a^*∗*^	4.3 ± 1.7
T5	8.27 a^*∗∗*^, b^*∗∗*^, c^*∗∗∗*^, d^*∗*^, e^*∗∗*^ 90.42±	1.5 ± 0.09 a^*∗∗*^, b^*∗∗*^, c^*∗∗*^, d^*∗*^, e^*∗∗*^

All values are presented as (Mean ± SEM). ^*∗*^*P* < 0.05, ^*∗∗*^*P* < 0.01, and ^*∗∗∗*^*P* < 0.001 represent a, b, c, d, and e, respectively, compared with C, T1, T2, T3, and T4 groups.

**Table 2:** Changes of the time spent and frequency of entry into the closed arms in different groups.

Groups	Time spent in closed arms (sec)	Number of entries in closed arms
C	175 ± 5.32	2.02 ± 0.5
T1	159.68 ± 8.19 a^*∗*^	2.7 ± 1.02
T2	149.22 ± 10.12 a^*∗∗*^	1.8 ± 1.1 a^*∗*^, b^*∗*^
T3	170.9 ± 7.23 b^*∗*^, c^*∗∗*^	2.4 ± 1.2 c^*∗*^
T4	158.03 ± 7.18 a^*∗*^	2.3 ± 0.9
T5	6.20 a^*∗∗*^, b^*∗∗*^, c^*∗∗∗*^, d^*∗*^, e^*∗∗*^ 209.58±	3.5 ± 0.04 a^*∗∗*^, b^*∗∗*^, c^*∗∗*^, d^*∗*^, e^*∗*^

All values are presented as (Mean ± SEM). ^*∗*^*P* < 0.05, ^*∗∗*^*P* < 0.01, and ^*∗∗∗*^*P* < 0.001 represent a, b, c, d, and e, respectively, compared with C, T1, T2, T3, and T4 groups.

**Table 3:** Proinflammatory cytokines concentration in serum of the animal studied.

	IL-1 (pg/ml)	TNF*α* (pg/ml)
C	98.45 ± 8.90	100.5 ± 12.5
T1	87.34 ± 12.56	95.2 ± 14.63
T2	76.54 ± 14.29	79.65 ± 8.93 a^*∗*^, b^*∗*^
T3	145.53 ± 11.24 a^*∗∗*^, b^*∗∗*^, c^*∗*^	120.34 ± 23.56 a^*∗*^, c^*∗*^
T4	128.69 ± 18.69 a^*∗*^, b^*∗*^, c^*∗*^	108.89 ± 14.67 d^*∗*^
T5	198.47 ± 23.12 a^*∗∗∗*^, b^*∗∗∗*^, c^*∗∗*^, d^*∗∗*^, e^*∗∗*^	253.56 ± 10.46 a^*∗∗∗*^, b^*∗∗∗*^, c^*∗∗∗*^, d^*∗∗*^, e^*∗∗*^

All values are presented as (mean ± SEM). ^*∗*^*P* < 0.05, ^*∗∗*^*P* < 0.01, and ^*∗∗∗*^*P* < 0.001 represent a, b, c, d, and e, respectively, compared with C, T1, T2, T3, and T4 groups.

**Table 4:** Grading of necrosis and gliosis in brain tissue.

	Score		C	T1	T2	T3	T4	T5
*Neuron necrosis*	Not seen	0	0	0	0	3	2	3
	Less than 25%	1						
	Between 25 and 50%	2						
	Between 50 and 75%	3						
	More than 75%	4						

*Gliosis*	Not seen	0	0	0	0	0	0	1
	Focal and less than 25%	1						
	Focal and between 25 and 50%	2						
	Focal but severe	3						
	Severe and diffuse	4						
Total score		0–8	0	0	0	3	2	4

**Table 5:** Mean ± SEM of counted neurons in the cerebral cortex of animals.

Group	Cortex
Control	81.88 ± 3.56
T1	82.2 ± 5.48
T2	82.49 ± 8.91
T3	52.8 ± 7.54 a^*∗*^, b^*∗*^, c^*∗∗*^
T4	69.83 ± 6.79 a^*∗*^, b^*∗*^, c^*∗*^, d^*∗*^
T5	36.60 ± 6.78 a^*∗∗∗*^, b^*∗∗∗*^, c^*∗∗∗*^, d^*∗∗*^, e^*∗*^

^*∗*^ *P* < 0.05, ^*∗∗*^*P* < 0.01, and ^*∗∗∗*^*P* < 0.001 represent a, b, c, d, and e, respectively, compared with C, T1, T2, T3, and T4 groups.

## Data Availability

The data can be obtained upon request to the corresponding author.
